# Species Richness Patterns and Water-Energy Dynamics in the Drylands of Northwest China

**DOI:** 10.1371/journal.pone.0066450

**Published:** 2013-06-20

**Authors:** Liping Li, Zhiheng Wang, Stefan Zerbe, Nurbay Abdusalih, Zhiyao Tang, Ming Ma, Linke Yin, Anwar Mohammat, Wenxuan Han, Jingyun Fang

**Affiliations:** 1 Department of Ecology, College of Urban and Environmental Sciences, Key Laboratory for Earth Surface Processes of the Ministry of Education, Peking University, Beijing, China; 2 Center for Macroecology, Evolution and Climate, Department of Biology, University of Copenhagen, Copenhagen, Denmark; 3 Faculty of Science and Technology, Free University of Bozen-Bolzano, Bolzano, Italy; 4 College of Resources and Environmental Sciences, Key Laboratory of Oasis Ecology of the Ministry of Education, Xinjiang University, Urumqi, China; 5 Xinjiang Institute of Ecology and Geography, Chinese Academy of Sciences, Urumqi, China; 6 Key Laboratory of Plant-Soil Interactions, Ministry of Education, College of Resources and Environmental Sciences, China Agricultural University, Beijing, China; Lakehead University, Canada

## Abstract

Dryland ecosystems are highly vulnerable to climatic and land-use changes, while the mechanisms underlying patterns of dryland species richness are still elusive. With distributions of 3637 native vascular plants, 154 mammals, and 425 birds in Xinjiang, China, we tested the water-energy dynamics hypothesis for species richness patterns in Central Asian drylands. Our results supported the water-energy dynamics hypothesis. We found that species richness of all three groups was a hump-shaped function of energy availability, but a linear function of water availability. We further found that water availability had stronger effects on plant richness, but weaker effects on vertebrate richness than energy availability. We conducted piecewise linear regressions to detect the breakpoints in the relationship between species richness and potential evapotranspiration which divided Xinjiang into low and high energy regions. The concordance between mammal and plant richness was stronger in high than in low energy regions, which was opposite to that between birds and plants. Plant richness had stronger effects than climate on mammal richness regardless of energy levels, but on bird richness only in high energy regions. The changes in the concordance between vertebrate and plant richness along the climatic gradient suggest that cautions are needed when using concordance between taxa in conservation planning.

## Introduction

Large-scale geographical patterns in species richness and the underlying mechanisms are among the central issues of biology [1]–[4]. They continue to attract scientific debate and drive the emergence of hypotheses to test them [5]–[6]. Many hypotheses were proposed in the last three decades, among which water-energy dynamics hypothesis [7]–[9] mechanistically links species richness patterns with climates. Evidence of climate effects on species richness has been shown in many regions and different ecosystems [10]–[12]. However, studies on species richness in drylands are extremely scarce.

Contemporary climate is considered as one of the crucial impacts on species richness patterns [11]. In particular, the water-energy dynamics hypothesis is widely tested for the patterns of plant species richness [7]–[8]. Variable relationships between species richness and energy availability, which is widely represented by potential evapotranspiration (PET), have been observed in previous studies [1], [10]. Species richness co-varies strongly with energy under cold climates, but the relationships become much more variable under warm climates [4], [11], [13]. According to the water-energy dynamics hypothesis, one can expect that species richness in arid regions is negatively correlated with energy availability, but positively correlated with water availability [4], [8], [11]. However, the contrasting impacts of water and energy on species richness in drylands remain poorly tested.

Plants are the primary energy source for terrestrial animals, providing energy directly for herbivores and frugivores and indirectly for carnivores via energy flow through the food web [14]. Moreover, terrestrial plants generate diverse habitats, and hence may benefit the speciation of animals via specialized plant-animal interactions or allopatric speciation [15], [16]. Therefore, terrestrial plants have long been considered as a potential determinant of terrestrial animal species richness. Previous studies indicated that species richness varied in parallel among plants and vertebrates [14]–[15], [17]–[20], among vertebrates [21], [22], among prey species and carnivore species [23], and among plants/vertebrates and hyperdiverse insect taxa [24]. Strong concordance between the species richness patterns of different groups could make conservation activities more cost effective. However, the concordance among the species richness of different taxa has rarely been tested in arid Central Asia due to a lack of data. Moreover, whether the concordance among taxa is consistent along climatic gradients remains poorly understood. The relative importance of plants and climate and their interactions in shaping animal species richness patterns are still controversial [18], [25].

Biologists rely on distribution maps of wildlife for conservation planning [26]. Unlike vertebrates, range maps for vascular plants are often less considered when planning protected areas [27]. This is because precise large-scale range maps of vascular plants are lacking [11], [28]. The global vascular plant species richness was plotted in 2005 with large amounts of data which is a big step forward for the mapping of global vascular plants [29]. However, the data quality and suitability in arid Central Asia, especially in the Xinjiang Uygur Autonomous Region (Xinjiang afterwards) in China, were poor. Drylands (i.e. arid, semi-arid and arid dry-subhumid regions) cover more than 40% of the terrestrial area and the ecosystems in drylands are highly vulnerable to rapid global climate change and desertification [30]. Therefore, studies on species richness patterns in the drylands in Central Asia are urgent. Moreover, although vertebrate species have been comprehensively investigated in Xinjiang [31]–[32], studies combining both plants and animals in this region have not yet been reported.

In this study, using a database of the distributions of vascular plants, mammals, and birds in one of the most arid regions in Central Asia (Xinjiang in northwest China), we mapped the species richness patterns of these three groups. Xinjiang contains the driest desert in China and also three large mountain ranges. From lowlands to high mountains, the vegetation changes from deserts to grasslands, forests and then to alpine meadows [33]. Xinjiang is well suited for this study because of its unique arid habitats, broad climatic gradients, and rich flora and fauna. In this study, we (1) tested the water-energy dynamics hypothesis on large-scale species richness patterns in one of world’s largest drylands; (2) evaluated the concordance among the species richness of the three groups, and the influence of water-energy dynamics on the concordance; (3) compared the relative importance of climate and plants in shaping the species richness of mammals and birds.

## Data and Methods

### Study Area

**Figure 1:**
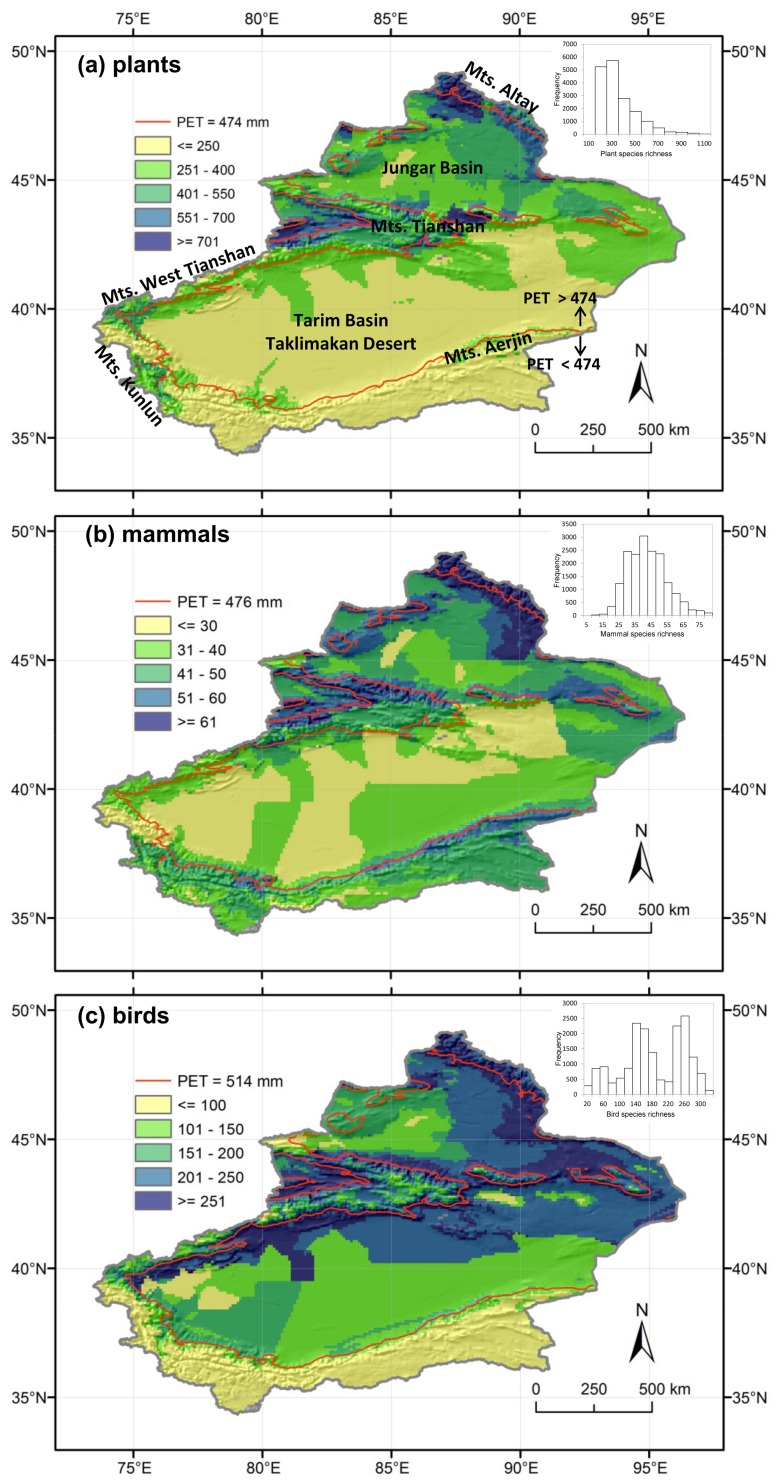
The patterns and frequency distributions of the species richness of plants (a), mammals (b), and birds (c) in Xinjiang, China. The grain size is 0.1°×0.1°. Lines in the figures show the PET values of 474, 476 and 514 mm which are the breakpoint values for the relationships of PET and plant, mammal and bird species richness.

Xinjiang of China, one of the world’s largest drylands, is located in Central Asia (see inset of Figure S1a in File S1), and covers 1.64 million km^2^ (more than 6 times the size of British). Geographically, Xinjiang is divided into two basins by the longitudinally orientated Tianshan Mountains, with the Jungar Basin in the north and the Tarim Basin in the south. The most northern and southern parts are the Altay Mountains and the Kunlun Mountains, respectively (Figure 1a, Figure S1a in File S1). The altitude ranges from 156 m below to 8611 m above sea level.

The climate is continental and varies greatly cross space. The precipitation is extremely scarce in most areas. The mean annual precipitation in the entire Xinjiang is 188 mm and the mean annual temperature is 10.4°C in the last 40 years (data from 56 climate stations in Xinjiang, China Meteorological Administration). The temperature extreme ranges from −51.5°C to 47.6°C geographically [33].

Due to the unique mountain-basin system, Xinjiang has a rich flora, and is very important for biodiversity conservation. For example, Xinjiang has 295 endemic plants, 129 key protected plants, 203 ephemeral plants, and 320 halophytes [34]. Western Tianshan Mountains in Xinjiang have been identified as a biodiversity hotspot of China for both plants and animals [35]–[36].

Both floras and faunas in Xinjiang have been well surveyed. For example, a large number of field surveys since the 1980 s have built a complete species list of vascular plants, which is 30% more than the species number prior to 1978 [37]–[38]. However, there have been no large-scale studies about the species richness patterns based on all vascular plants, mammals, and birds.

### Species Distribution Data

Xinjiang is home for 3637 native vascular plant species belonging to 746 genera and 121 families, 154 mammal species belonging to 23 families and 7 orders, and 425 bird species belonging to 56 families and 21 orders. The distributions of vascular plants were compiled from the Xinjiang Ecological Resources and the Environment Database (from http://www.csdb.cn) and the Flora of Xinjiang [38]. The distributions of bird and mammal species were from reference [31]–[32], respectively. These data sources reflect the most current collection of plants and vertebrates in this region. We did not include the reptiles and amphibians in the analyses because these taxa have extremely low species richness (40–45 of reptiles and 9 of amphibians) in our study area.

The species distribution information in the literature was mainly recorded at county level. The counties in Xinjiang, especially those in southern Xinjiang, are relatively big (e.g. Ruoqiang County with 199,000 km^2^ in size). The actual distribution of a species in a big county may be restricted in a part of it according to the environmental requirement (particularly altitude in our study area) of the species. To improve the quality of the distribution data, we first gridded the entire region into 0.1°×0.1° grids, and then overlaid the grids on the topography of Xinjiang to estimate the altitudinal ranges of each grid cell. Second, we overlaid the county level distributions of species with the grids of Xinjiang. Third, we removed the grid cells whose altitude ranges fell out of the altitudinal ranges of the species. Totally, we collected altitudinal ranges of 62% of the plant species, 43% of the mammal species and 85% of the bird species. On average, this method reduced the distribution area of plants, birds, and mammals by 28%, 27%, and 25%, respectively, from the county-level distributions to the gridded distributions (see Figure S2 in File S1 for examples). The data quality was evaluated by local botanists (see S3 in File S1 for detail).

The topographical data with a resolution of 0.0083 arc degree (equivalent to 1 km at the equator) was obtained from the United States Geological Survey (USGS) (Figure S1a in File S1). We repeated the transformation from county-level to gridded distributions using grids with resolutions of 0.5°×0.5° and 1.0°×1.0°, and found that the species richness patterns (Figure S4 in File S1), their relationships with climate and the concordance between different groups (Figures S5-S6 in File S1) were all consistent with those at the resolution of 0.1°×0.1°. Therefore, we reported only the results at the resolution of 0.1°×0.1° for concision, and included the results at resolutions of 0.5°×0.5° and 1.0°×1.0° in the Supporting Files (Figure S4 and Tables S5-S6 in File S1).

### Climatic Data

The climatic data with a resolution of 0.0083 arc degree were obtained from the World Climate Database [39]. This database contains the global monthly temperature and precipitation, which were used to calculate the PET and actual annual evapotranspiration (AET) [40]. PET was widely used to represent the energy availability of a region [1]. In Xinjiang, AET reflects the amount of water that plants can actually use. It is strongly linearly correlated with precipitation, and hence was used to represent water availability. PET in this region is higher in basins than in mountains but AET is higher in mountains than in basins (see Figure S1b,c in File S1). PET and AET are not correlated with each other and hence will not introduce multi-collinearity in the following regression analysis (see Figure S1d in File S1). Other climatic variables were also analysed, including mean annual temperature (MAT), growing season temperature (GST), mean temperature of the coldest month (MTCM) and the warmest month (MTWM), annual biological temperature (ABT), warmth index (WI), coldness index (CI), annual precipitation (AP), growing season precipitation (GSP), water deficiency (WD), and moisture index (Im) [5]. However, because of the high collinearity between these variables, we selected only AET and PET in the following analyses.

### Data Analyses

First, ordinary least square (OLS) regressions were conducted to analyze the relationships between species richness and climatic variables. As strong hump-shaped relationships between species richness and PET were observed for all the three groups, we used piecewise linear regressions to explore these relationships. Piecewise linear regression attempts to find the heterogeneity of the relationship between two variables by identifying one or more breakpoints, and then fits linear regressions for each piece before and after the breakpoints [41]. The significance of the breakpoints was tested using F tests [41]. For the groups with significant breakpoints in the richness-PET relationships, we compared the *R^2^* and slopes of the richness-AET relationships between the low and high energy regions (defined as before and after PET breakpoints of plants, mammals, and birds, respectively). We evaluated the slope differences between low and high energy regions using analysis of covariance (ANCOVA) with the following model: species richness = a+b × AET+c × region+d × (AET*region), where region is a factor variable representing high energy region (1) *vs.* low energy region (0), a, b, c and d are regression coefficients, and AET*region represents the interaction between AET and region. Significant AET*region interaction means that the slopes of AET on species richness are significantly different between low and high energy regions. Spatial autocorrelation in gridded ecological data is generally strong (Figure S7 in File S1), which could inflate the Type *I* error in statistical tests and lead to more significant results than data actually support [42]. To account for the effects of spatial autocorrelation, we tested the significance of the AET*region interaction in ANCOVA using bootstrap method with the following three steps [43]: (1) Randomly sampled 5% of all grid cells from the entire dataset. A threshold of 5% was used because the average distance between the randomly sampled grid cells is c.a. 700 km, which corresponds to the distance at which the spatial autocorrelation in plant and bird richness becomes 0 and that in mammal richness becomes slightly negative. (2) ANCOCA was conducted using the random sample, and the *p* value of the interaction was estimated. (3) Step 1 and 2 were repeated for 1000 times. Only when more than 95% of random samples generated significant interactions, the slope difference was considered significant.

Second, multiple OLS regressions were conducted to explore the combined effects of PET and AET on species richness. For PET, we used a × PET+b × PET^2^ in the regression for the entire region because of the hump-shaped functions between species richness and PET. To compare the relative effects of different climatic variables on species richness, we conducted partial regressions with species richness as response variable, and AET and PET as predictors. Partial regression could divide the total variance in species richness into: (i) pure effects of AET and PET; (ii) co-varying effects between AET and PET; and (iii) unexplained variance [44].

Third, simple OLS regressions were used to evaluate the concordance between bird or mammal species richness and plant richness. Similarly, ANCOVA in combination with bootstrap method was used to evaluate the difference in the species richness concordance between low and high energy regions: bird (or mammal) richness = a+b × plant richness+c × region+d × (plant richness*region). Significant plant richness*region interaction means that the concordance between bird (or mammal) richness and plant richness is significantly different between low and high energy regions. Partial regressions were also used to compare the relative effects of plant species richness and climate on the species richness of mammals and birds. We used a × PET+b × PET^2^+ c × AET to represent climate in the regression for the entire region, and a × PET+b × AET for either low or high energy regions. Here we defined the low and high energy regions as before and after the PET breakpoint of plant species richness. We also repeated the analyses with the breakpoints of mammals and birds and the results were similar. In partial regressions, the multi-collinearity between plant richness and climate may potentially reduce the performance of the combined model with both climate and plant richness as predictors. Therefore, we estimated the Variance Inflation Factor (VIF) of plant richness in all partial regressions [44]. Generally, VIF values greater than five suggest significant effects of multi-collinearity.

Fourth, to further explore the direct and indirect effects of climate and plant richness on bird and mammal richness, we conducted structural equation modeling (SEM) analysis. SEMs allow *a priori* hypothesized causal relationships between dependent variables and predictors (which is also called path models), and test these hypothesized relationships by partitioning the correlations between dependent variables and predictors into direct and indirect effects [45]. Direct effects are measured by the standardized partial regression coefficients between dependent variables and predictors, and indirect effects by the products of all direct effects over the paths linking the dependent variables and predictors. SEMs have been widely used to explore the richness concordance between different groups [14], [16], [20]. Here, we developed *a priori* SEM with hypothesized direct links between bird (or mammal) richness and PET, AET and plant richness, and between plant richness and PET, AET.

All the analyses were conducted with R 2.12.2 [46]. In particular, piecewise regression was conducted with the R scripts developed by [41], and SEM analysis with R package “sem” (http://socserv.socsci.mcmaster.ca/jfox/). The species richness data in the three resolutions is available as File S2.

## Results

### Species Richness Patterns

Plant species richness was higher in the north than in the south and in the mountains than in the basins. The highest plant richness occurred in the western Altay Mountains and the western and central parts of the Tianshan Mountains (Figure 1a). Mammal species richness was higher in mountains than in basins. There were some ungulates distributed in low and middle altitudes of the Kunlun Mountains which made this area high in mammal species richness (Figure 1b). Bird species richness was high in the north (i.e. Altay Mountains) and the low to middle altitudes of the Tianshan Mountains in both northern and southern ridges (Figure 1c).

### Climatic Determinants of Species Richness Patterns

**Figure 2:**
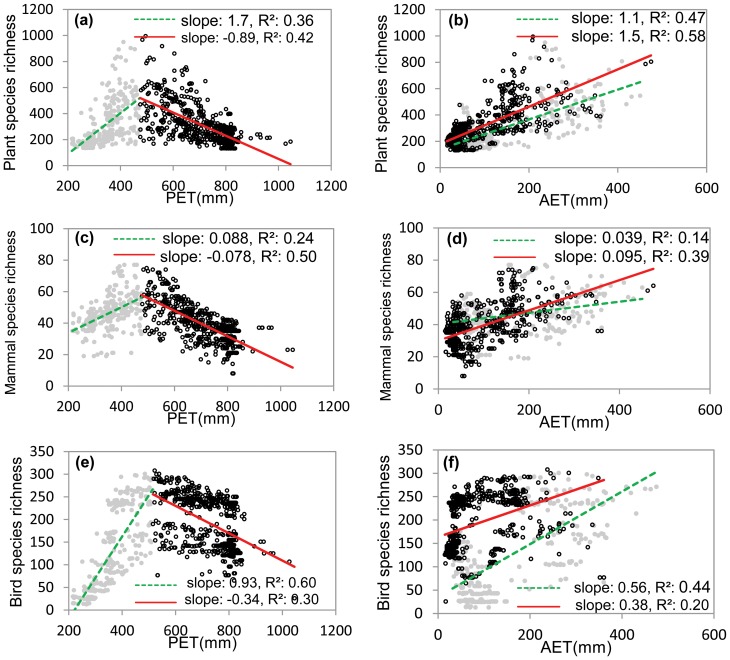
Changes in species richness with potential evapotranspiration (PET) and actual evapotranspiration (AET) for plants (a, b), mammals (c, d), and birds (e, f). The grey filled dots and dashed lines represent the relationships in the low energy region, while the black open dots and solid lines represent the relationships in the high energy region. Figures show 5% randomly sampled data.

Species richness of vascular plants, mammals, and birds was all observed to have hump-shaped functions of PET: species richness first increased, and then decreased with increasing PET, suggesting that energy availability stimulated species richness when it was low but suppressed species richness when it was high. The breakpoints of the relationships were at PET = 474, 476, and 514 mm for vascular plants, mammals, and birds, respectively (Figure 2a,c,e). In the entire Xinjiang and both low and high energy regions, species richness of the three groups increased linearly with increasing AET (Figure 2b,d,f), indicating that water availability always stimulated species richness. Moreover, ANCOVA analysis indicated that the slopes of richness-AET relationships for plants and mammals were significantly (or marginally significantly) higher in high than low energy regions (for plants, slopes = 1.12 and 1.46 in low and high energy regions, *p* = 0.052; for mammals, slopes = 0.04 and 0.10 in low and high energy regions, *p*<0.001; Figure 2b,d), whereas the slopes of bird richness-AET relationships were significantly lower in high than in low energy regions (slopes = 0.56 and 0.38 in low and high energy regions, *p* = 0.014, Figure 2f). The differences in the slopes of richness-AET relationships suggest that species richness increased faster with increasing AET in the high energy region than in the low energy region for plants and mammals,but slower in the high energy region than in the low energy region for birds.

**Figure 3:**
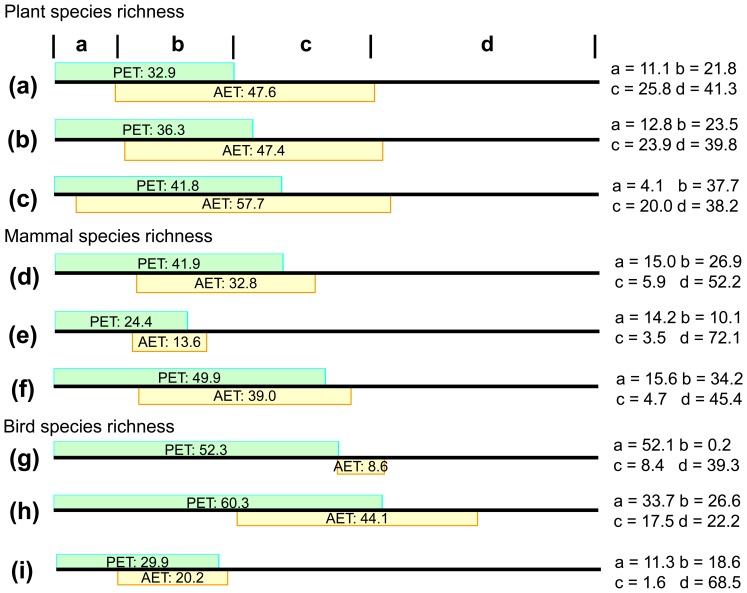
Comparisons between the effects of actual evapotranspiration (AET) and potential evapotranspiration (PET) on the species richness of plants, mammals, and birds in the entire Xinjiang region (a, d, g), and in the low (b, e, h) and high (c, f, i) energy regions by partial regressions.

Multiple OLS regressions indicated that AET and PET together explained 58.7%, 47.8%, and 60.7% of the variances in richness of plants, mammals, and birds in the entire region (Table S5 in File S1). The explanatory power was 60.2%, 27.9%, and 77.8% in the low energy region for the three groups, while it was 61.8%, 54.6%, and 31.5% in the high energy region (Table S5 in File S1). Partial regressions indicated that pure PET and AET effects were 11.1% and 25.8% respectively on the plant species richness in the entire Xinjiang. Similarly, the pure PET effect on plant richness was lower than pure AET effect in both low and high energy regions (Figure 3, Table S5 in File S1). In contrast to plants, the pure PET effect on vertebrate species richness (15.0% and 52.1% for mammal and bird species richness) was higher than pure AET effect (5.9% and 8.4% for mammal and bird species richness) in the entire Xinjiang and in both low and high energy regions separately (Figure 3, Table S5 in File S1).

### Concordance among Vascular Plants, Mammals and Birds

**Figure 4:**
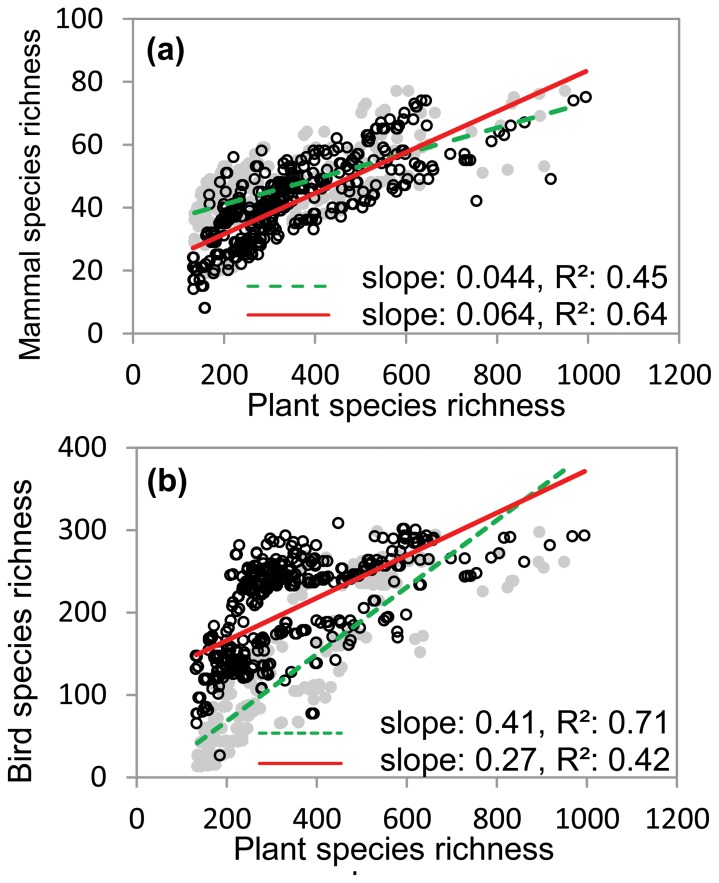
Concordance among plant and vertebrate species richness in Xinjiang, China. The grey filled dots and dashed lines represent the relationships in the low energy region, while the black open dots and solid lines represent the relationships in the high energy region. Figures show 5% randomly sampled data.

Both mammal and bird species richness was strongly correlated with that of plants (Figure 4). In general, plant richness was more strongly correlated with mammal richness (*R^2^* = 0.54) than with bird richness (*R^2^* = 0.38). Moreover, ANCOVA indicated that the correlation between plants and vertebrates significantly varied along the energy gradient. Particularly, the correlation between mammal and plant richness was weaker in the low energy region than in the high energy region (*R^2^* = 0.45 and 0.64, and slopes = 0.044 and 0.064 for the low and high energy regions, respectively, *p* = 0.001; Figure 4a). In contrast, the correlation between bird and plant richness was stronger in the low energy region than in the high energy region (*R^2^* = 0.71 and 0.42, slopes = 0.41 and 0.27 for the low and high energy regions, respectively, *p*<0.001; Figure 4b).

### The Effects of Climate vs. Plants on Species Richness of Mammals and Birds

Plant species richness and climate together explained 66.3% and 70.0% of the variances in richness of mammals and birds in the entire Xinjiang (Table S6 in File S1). They explained 47.7% and 84.5% of the variances in mammal and bird species richness in the low energy region, and 70.5% and 45.1% in the high energy region, respectively. This suggested that plant species richness and climate explained more variances of mammal species richness but less of bird species richness in the high energy region than in the low energy region.

**Figure 5:**
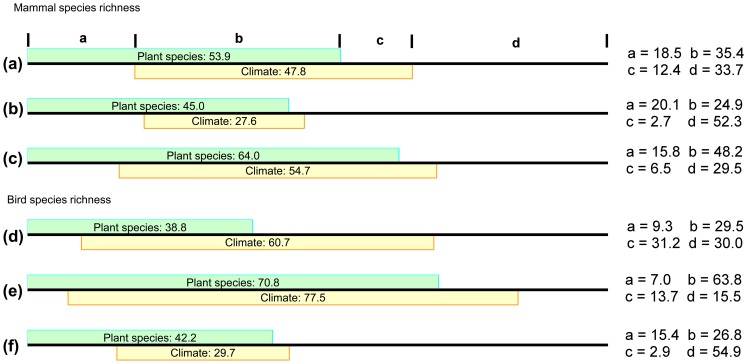
Comparisons between the effects of plant species richness and climate on species richness of mammals and birds in the entire Xinjiang region (a, d), and in low (b, e) and high (c, f) energy regions by partial regressions.

Partial regressions indicated that the pure effect of plant richness on mammals was stronger than the pure climatic effect in the entire Xinjiang and in both low and high energy regions (Figure 5 and Table S6 in File S1; entire Xinjiang: 18.5% *vs.* 12.4%; low energy region: 20.1% *vs.* 2.7%; high energy region: 15.8% *vs.* 6.5%). In contrast, the pure effect of plant richness on bird richness was weaker than the pure climatic effect in the entire Xinjiang and the low energy region (Figure 5 and Table S6 in File S1; entire Xinjiang: 9.3% *vs.* 31.2%; low energy region: 7.0% *vs.* 13.7%), but higher in the high energy region (Figure 5 and Table S6 in File S1; 15.4% *vs.* 2.9%). It is noteworthy that the co-varying effects between plant richness and climate are strong. In this study, the variance inflation factors (VIFs) of plant richness were 2.4, 2.5 and 2.6 in the multiple models of bird richness in entire Xinjiang, and in low and high energy regions respectively, and were 2.4, 2.4 and 2.7 in the multiple models of mammal richness. The VIF values suggested that multi-collinearity did not significantly bias the model performance of multiple regressions.

**Figure 6:**
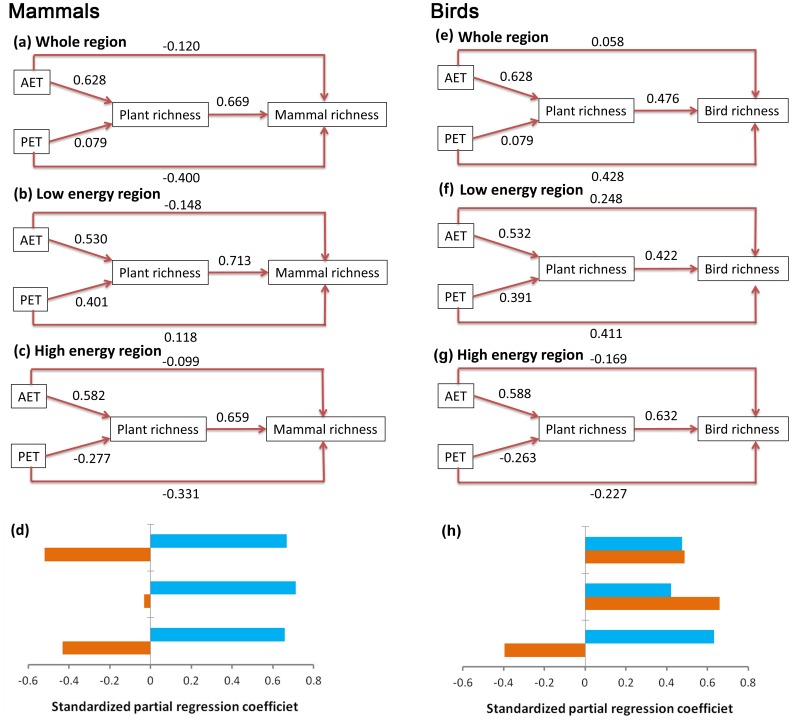
Structural equation models (SEM) of vertebrate and plant richness and climatic predictors. SEMs were conducted for mammals (a, b, c) and birds (e, f, g) in the entire Xinjiang (a, e) and in the low (b, f) and high (c, g) energy regions, respectively. (d) and (h) show the comparisons between the effects of plant richness (blue bars) and the total direct effects of climate (AET+PET) (orange bars) on vertebrate richness in the entire Xinjiang, low and high energy regions, respectively.

The results of SEMs (Figure 6) were consistent with those of OLS and partial regressions. First, SEMs indicated that plant richness had stronger direct effects on vertebrate richness than both AET and PET in entire Xinjiang and low and high energy regions (Figure 6). The effects of plant richness were also stronger than the total direct effects of climate on mammal richness regardless of energy levels (Figure 6d) and on bird richness in the high energy region (Figure 6h). The total direct effects of climate were comparable with, or stronger than, those of plant richness on bird richness in entire Xinjiang and the low energy region. More interestingly, AET showed much stronger indirect effects via plant species richness than direct effects on both mammal and bird richness regardless of energy levels, except in the case of bird richness in the low energy region where the direct effects of AET were slightly higher than its indirect effects. In contrast, PET showed much lower indirect than direct effects on both mammal and bird richness regardless of energy levels except in the case of mammal richness in the low energy region where the indirect effects were stronger.

## Discussion

### Species Richness Patterns and the Climatic Impacts

Species richness is higher in mountains than in basins in Xinjiang, which is consistent with previous findings at regional (e.g. in China [47]) and global scale [48]. The Altay Mountains and the Western Tianshan Mountains, with a favored climate in drylands (i.e. relatively high precipitation and moderate temperature), have been identified as hotspots of plants, mammals, and birds in China. The ecosystems in these two regions are listed in the ‘Important areas for conserving China’s priority ecosystems’ [49] because they are home to many unique species. For example, the Western Tianshan Mountains harbor many fruit plants that are the ancestors of modern cultivated fruits (e.g. *Malus sieversii*, *Armeniaca vulgaris*, *Prunus sogdiana*, and *P. domestica*) [35], [50] and bird species with high protection grades, for example, waterfowl and raptors [36].

The Taklimakan Desert in the Tarim Basin of southern Xinjiang is the largest desert in China, the second largest desert and one of the most arid regions in the world [51]. The extreme arid conditions lead to very low species richness, especially plants (Figure 1a). But the low species richness makes the species diversity in the drylands more important and should be given even higher priority because the ecosystems are highly vulnerable to global environmental change and desertification [30]. The drylands in Xinjiang harbor many species endemic to arid environment. For example, 19 plant species are endemic to the Taklimakan Desert in the Tarim Basin in southern Xinjiang and 24 to the Gurbantunggut Desert in the Jungar Basin in northern Xinjiang [52]. These endemic species have unique adaptations to extreme environments, are important for understanding the evolution of draught tolerance, and hence should be taken into consideration in the conservation planning in drylands.

Our findings on the richness-climate relationships are consistent with the predictions of the water-energy dynamics hypothesis [4], [8]. However, the breakpoints of the richness-PET relationships found in our study are different from previous findings. A breakpoint at PET = 474 mm (corresponding to MAT = 3°C [53]) was found for plant richness-PET relationship in Xinjiang which was slightly lower than that found in the global scale plant richness-PET relationship (a breakpoint at PET = 505 mm) [11]. Similarly, the breakpoints in the richness-PET relationships of mammals and birds in Xinjiang were also lower than those found in other regions. For example, for the North American mammals, a breakpoint at PET = 1000 mm was found, which doubles the values found in Xinjiang (PET = 476 mm) [10]. Moreover, a breakpoint at PET = 525 mm was found in the species richness-PET relationship for North American birds [1]. The differences between these results are probably due to the drier climate in Xinjiang.

More interestingly, our results suggested that the relative effects of energy and water availability varied for plants and vertebrates. In the entire Xinjiang, our results suggested that plant species richness was more strongly limited by water availability, while vertebrate species richness was more strongly limited by energy availability. Similar results were also found in North America, where the richness of vertebrates (birds, mammals, amphibians, and reptiles) was more influenced by energy (represented by PET) while tree species richness was more influenced by water availability (represented by AET) [1]. Our results and similar previous findings suggest that plant and vertebrate richness might be determined by different processes. Water availability could strongly limit plant growth via its direct influences on plant physiological (e.g. photosynthesis) tolerance, especially in arid areas like Xinjiang. However, water might not directly influence vertebrate physiological processes and consequently vertebrate species richness. In contrast, water might more strongly influence vertebrate species via its influence on plants. Plants are food resources and habitats of many vertebrates (frugivores and carnivores) and they influence vertebrate richness via vertebrates’ dietary requirements or niches [14].

Moreover, our results suggested that the relative effects of energy and water availability did not vary along energy gradient. In both low and high energy regions of Xinjiang, water availability has stronger effects on plant richness than energy availability, while energy availability has stronger effects on vertebrate richness than water. In contrast to our findings, a previous study on global plant richness patterns showed that energy availability (represented by PET) was the primary determinant of plant species richness in low energy regions, while water availability (represented by the number of wet days) was the primary determinant in high energy regions [11]. The inconsistency between our and previous findings about the primary determinants of plant richness might be because our study was conducted in one of the driest places in the world, and the water availability in this region is much more limiting for plant growth than in other regions.

### Concordance among the Species Richness of Plants and Vertebrates

Large scale species richness concordance among different taxa has been found globally [19] and in China’s nature reserves [18], [20]. Consistent with previous studies, we also found strong concordance between vertebrate and plant species richness in the drylands in Xinjiang. One possible explanation for the strong concordance between vertebrate and plant species richness is that plants and vertebrates may have similar feedback to climate [25], [54]. However, after the effects of climate were accounted for, plant richness still had the strongest direct effects on both bird and mammal richness regardless of energy levels. Alternatively, the strong concordance between vertebrate and plant richness might be due to functional links between these two groups. First, plants could influence animal species via food resource-consumer link. Plants provide food resources for herbivores and frugivors for their survival, and higher food diversity provided by higher diversity of food plant species might directly lead to higher animal species richness. For example, the species richness of frugivore birds in Africa is primarily determined by the diversity of their food plants, *Ficus* [14]. Second, plant species may influence animals via habitat heterogeneity generated in plant communities, as high plant richness may lead to more complex community structures. For example, a recent study suggested that local forest structures significantly influenced the bird species richness in Canada [16]. However, the relative importance of biological co-dependence and environmental drivers on richness remains unclear [55]. Our results suggested that plants always had a stronger influence on mammal species richness than climate regardless of energy levels. In contrast, plants had a stronger effect than climate on bird species richness only in the high energy region.

Moreover, our results also suggested that the concordance patterns between vertebrates and plant richness varied between different vertebrate groups. Particularly, after accounting for the climatic effects, the concordance between mammal and plant richness is consistently stronger than that between bird and plant richness in the entire Xinjiang and both low and high energy regions. The concordance between plants and mammals *vs.* plants and birds showed opposing patterns in low and high energy regions. The difference between mammals and birds might be due to their different dependence on plants. In Xinjiang, most mammals are herbivores and frugivors, which are strongly dependent on plant seeds, fruits or biomass, and therefore may be more strongly correlated with plant richness. For example, northern Xinjiang is one of the regions with the highest rodent species richness, while the Kunlun Mountains in southern Xinjiang are one of the regions with the highest lagomorph species richness [56]. Both of these groups heavily rely on plants. In contrast, birds in this region are dominantly carnivores [36], and thus may have a weaker or indirect dependence on plants, especially in low energy region. Cross-taxon congruence patterns are complex [57], and the underlying mechanism of the concordance was tested [58] and still needs more tests in various regions and for different taxa.

### Conclusions

Our study adds to the limited knowledge of species richness patterns in the drylands of Central Asia, one of the driest regions of the world, and will benefit future conservation planning in this region. We found that plant richness is more strongly influenced by water availability while vertebrate richness is more strongly determined by energy availability. Energy variables influence vertebrate species richness more directly while the water variables impact vertebrate species richness via the effects of plants more than the direct effects. The variances in the concordance between vertebrate and plant species richness along energy gradient and across different groups suggest that cautions are needed when using concordance patterns between taxa in the conservation planning.

## Supporting Information

File S1 Figure S1, The topography, potential evapotranspiration (PET), actual evapotranspiration (AET), and the correlation between PET and AET in Xinjiang, China. Figure S2, The examples for the transformation from the county-level distributions to the gridded distributions by filtering out the regions where the altitudinal ranges fell out of the species altitudinal ranges. Figure S3, The explanation of the verification of the dataset. Figure S4, The patterns of the species richness of plants, mammals, and birds in Xinjiang, China. The grain sizes are 0.5°×0.5°and 1.0°×1.0°, respectively. Table S5, The effects of climate (PET and AET) on plant and vertebrate species richness in Xinjiang, China by partial regressions. The grain sizes are 0.1°×0.1°, 0.5°×0.5° and 1.0°×1.0°, respectively. Table S6, The effects of plant species richness and climate (PET and AET) on vertebrate species richness in Xinjiang, China by partial regressions. The grain sizes are 0.1°×0.1°, 0.5°×0.5° and 1.0°×1.0°, respectively. Figure S7, Spatial correlograms (estimated by Moran’s I coefficients) for the patterns in species richness (plants, mammals and birds) and the model residuals.
(DOCX)

File S2 **The data of species richness (plants, mammals and birds) in Xinjiang, China.** The grain sizes are 0.1°×0.1°, 0.5°×0.5° and 1.0°×1.0°, respectively.
(XLSX)
