# Supplementary material for: Species Richness Patterns and Water-Energy Dynamics in the Drylands of Northwest China
Source: PLoS One. 2013 Jun 20;8(6):e66450. doi: 10.1371/journal.pone.0066450 (PMC3688736; doi:10.1371/journal.pone.0066450)
Supplement: File S1 — Figure S1, The topography, potential evapotranspiration (PET), actual evapotranspiration (AET), and the correlation between PET and AET in Xinjiang, China. Figure S2, The examples for the transformation from the county-level distributions to the gridded distributions by filtering out the regions where the altitudinal ranges fell out of the species altitudinal ranges. Figure S3, The explanation of the verification of the dataset. Figure S4, The patterns of the species richness of plants, mammals, and birds in Xinjiang, China. The grain sizes are 0.5°×0.5°and 1.0°×1.0°, respectively. Table S5, The effects of climate (PET and AET) on plant and vertebrate species richness in Xinjiang, China by partial regressions. The grain sizes are 0.1°×0.1°, 0.5°×0.5° and 1.0°×1.0°, respectively. Table S6, The effects of plant species richness and climate (PET and AET) on vertebrate species richness in Xinjiang, China by partial regressions. The grain sizes are 0.1°×0.1°, 0.5°×0.5° and 1.0°×1.0°, respectively. Figure S7, Spatial correlograms (estimated by Moran’s I coefficients) for the patterns in species richness (plants, mammals and birds) and the model residuals. (DOCX) [file pone.0066450.s001.docx]

**Figure S1** The topography (a), potential evapotranspiration (PET) (b), actual evapotranspiration (AET) (c), and the correlation between PET and AET (d) in Xinjiang, China. The seven regions outlined on the map show roughly the mountains and basins of Xinjiang. The inset in (a) shows the location of Xinjiang in China. For (d), figure shows 5% randomly sampled data.

**
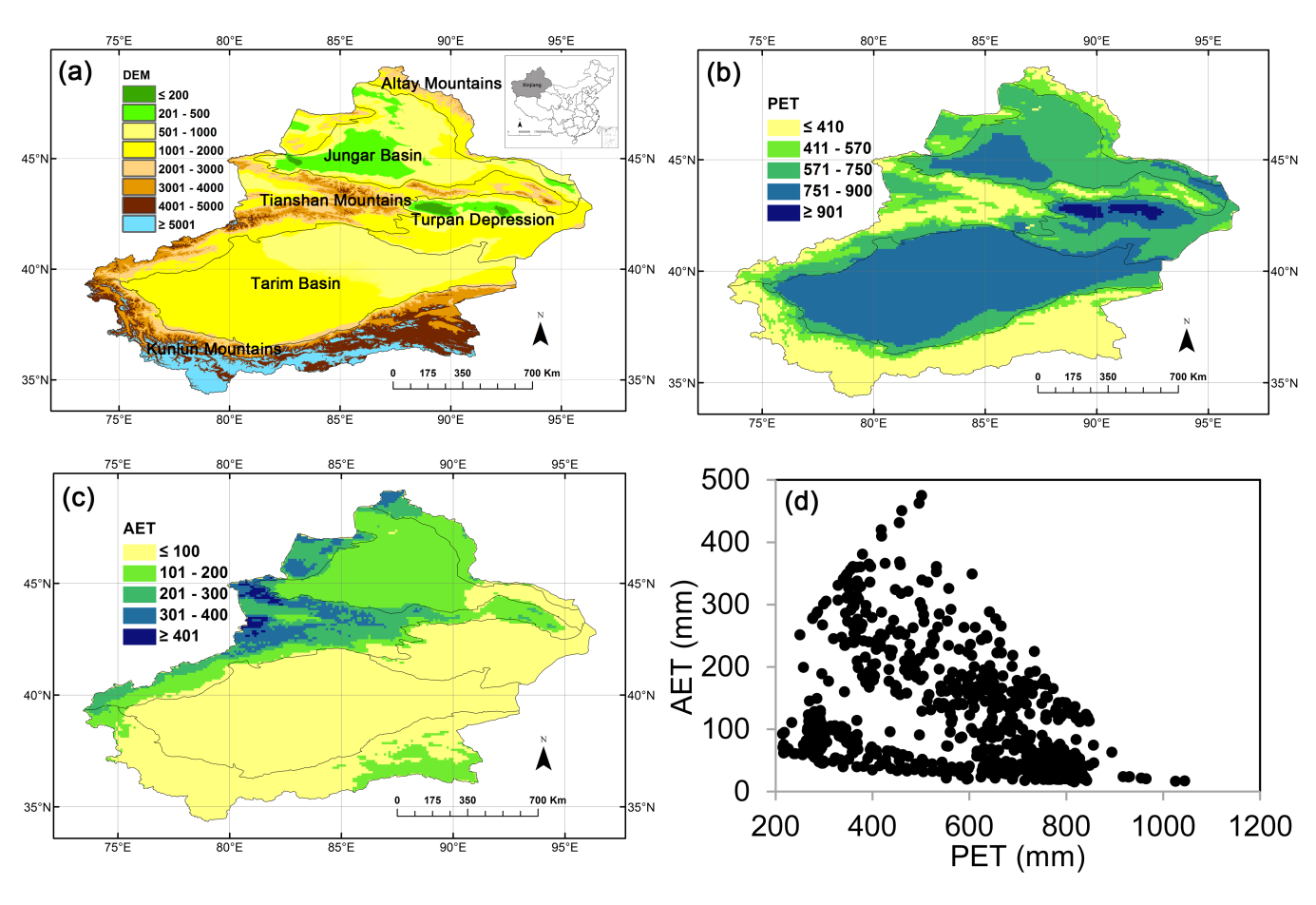
**

**Figure S2** The examples for the transformation from the county-level distributions (a, c) to the gridded distributions (b, d) by filtering out the regions where the altitudinal ranges fell out of the species altitudinal ranges. (a, b), *Picea schrenkiana*; (c, d), *Juniperus sibirica*. There are 9270 (before transformation) and 2483 (after transformation) grids for *P. schrenkiana* (73% less) and 10669 (before) and 3420 (after) grids for *J. sibirica* (68% less). The seven regions outlined on the map show roughly the mountains and basins of Xinjiang.

**
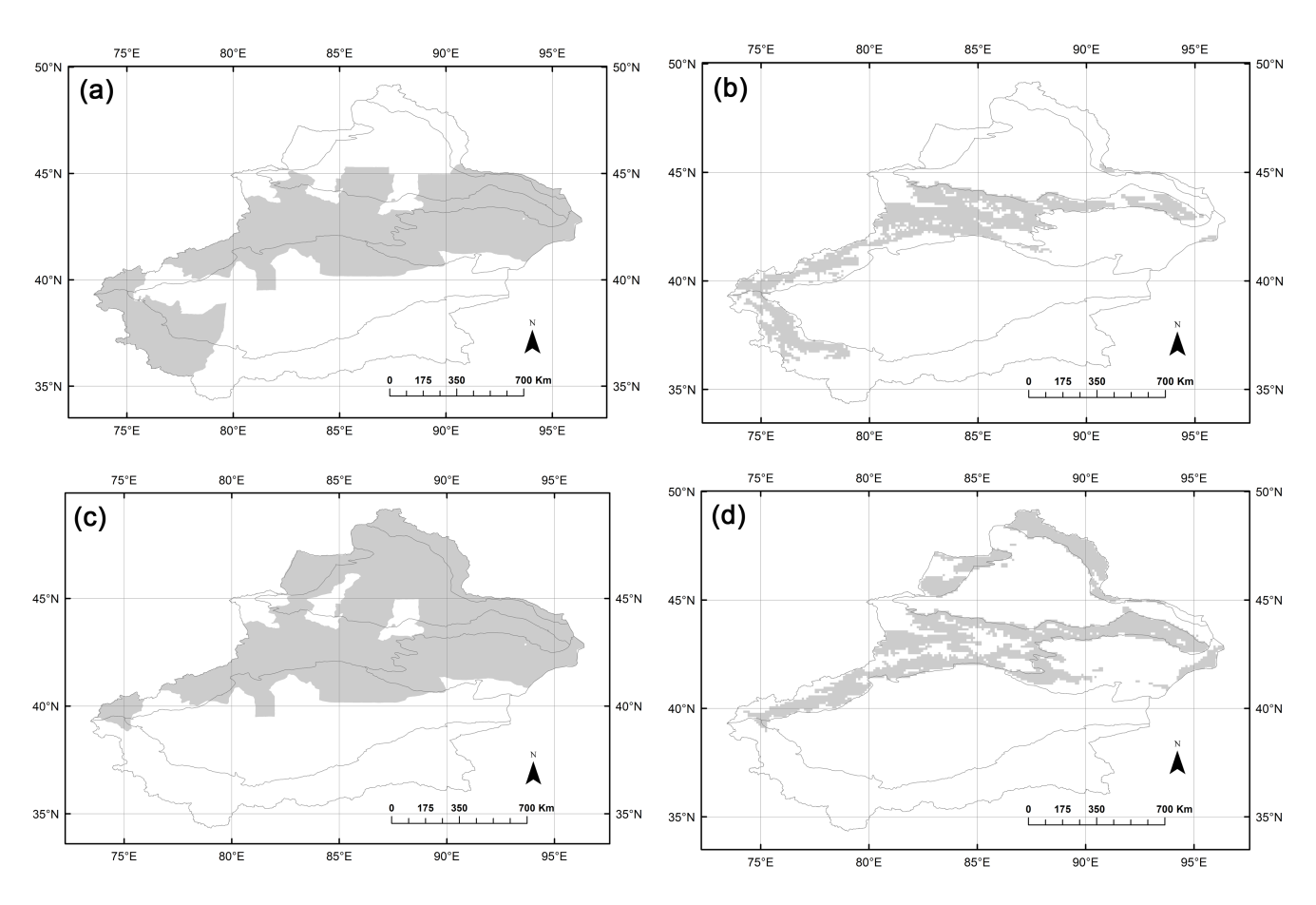
**

**S3** The verification of the dataset. Distribution maps of 41 plant species were randomly selected (about 1% of the total plant species) and sent to the local botanists to check the validity of our data. We overlaid the species distributions in our dataset and the suggested distributions of experts and found that the distribution areas of plants in our dataset were 11.4% less and 1.3% more than the suggestions of experts, respectively. This showed that the data was reliable for the further analysis. The 41 species were: *Cystopteris fragilis, Asplenium tianshanicum, Polystichum alatawshanicum, Abies sibirica, Juniperus jarkendensis, Ephedra intermedia, Ephedra equisetina, Salix neolapponum, Polygonum viviparum, Salicornia europaea, Halocnemum strobilaceum, Celosia argentea, Amaranthus retroflexus, Cerastium cerastoides, Dianthus superbus, Delphinium kunlunshanicum, Delphinium tarbagataicum, Berberis hetropoda, Sophora alopecuroides, Glycyrrhiza glabra, Glycyrrhiza inflata, Geranium collinum, Nitraria tangutorum, Polygala hybrida, Cortusa brotheri, Primula algida, Eritrichium aktonense, Dracocephalum integrifolium, Dracocephalum grandiflorum, Phlomis pratensis, Plantago depressa, Inula britanica, Senecio nemorensis, Saussurea cinerea, Phragmites australis, Poa angustifolia, Poa pratensis, Elytrigia repens, Leymus secalinus, Fritillaria tachengensis, Orchis umbrosa.*

**Figure S4** The patterns of the species richness of plants (a, b), mammals (c, d), and birds (e, f) in Xinjiang, China. The grain sizes are 0.5° × 0.5° (a, c, e) and 1.0° × 1.0° (b, d, f), respectively. Lines in the figures show the major rivers of Xinjiang.


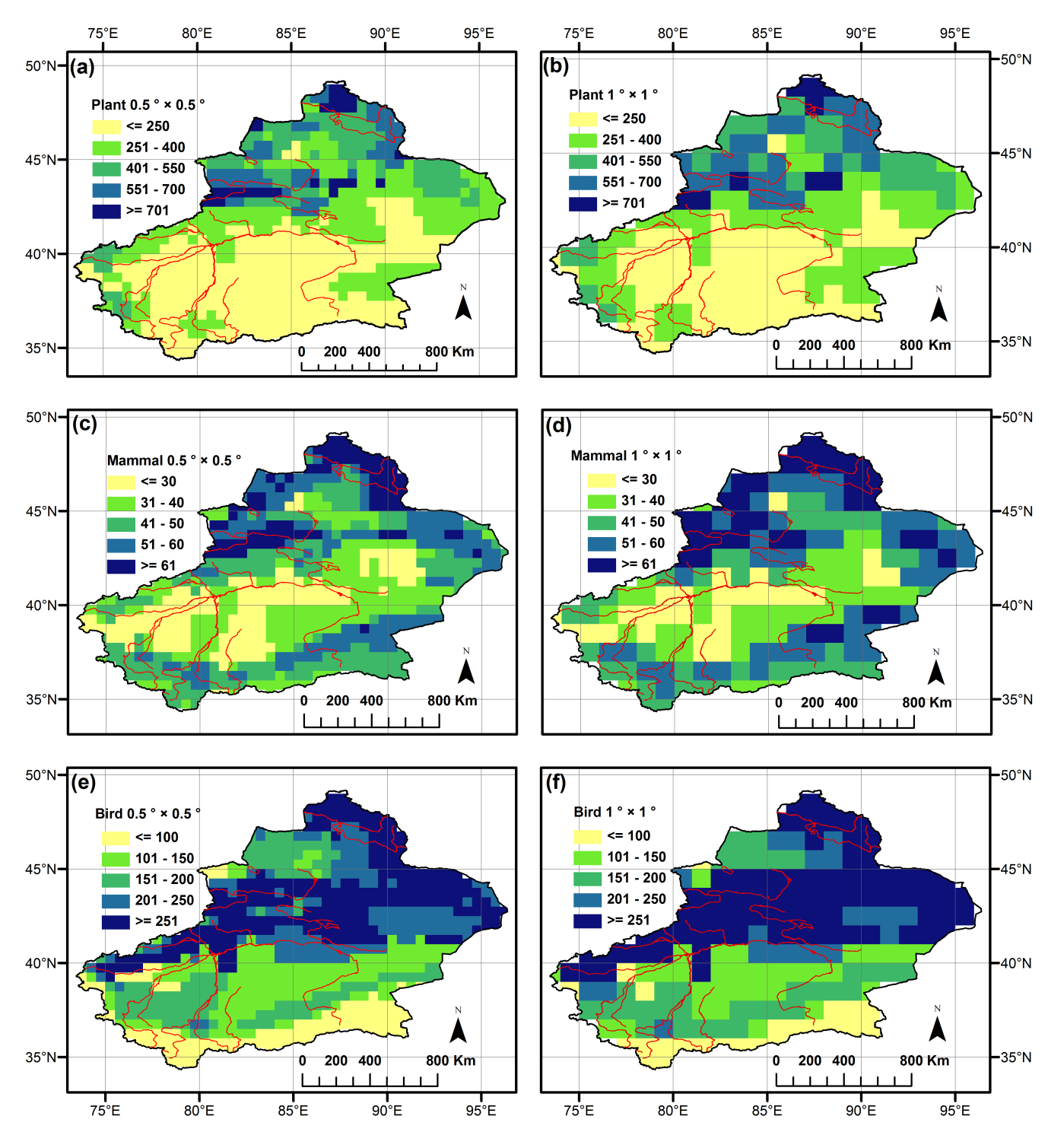


**Table S5** The effects of climate (PET and AET) on plant and vertebrate species richness in Xinjiang, China by partial regressions. The grain sizes are 0.1° × 0.1°, 0.5° × 0.5° and 1.0° × 1.0°, respectively. PET: potential evapotranspiration and AET: actual evapotranspiration.

|  |  |  | 0.1° × 0.1° |  |  | 0.5° × 0.5° |  |  | 1.0° × 1.0° |  |
| --- | --- | --- | --- | --- | --- | --- | --- | --- | --- | --- |
|  |  | Plant richness | Mammal richness | Bird richness | Plant richness | Mammal richness | Bird richness | Plant richness | Mammal richness | Bird richness |
| Whole region | Full model | 0.59 | 0.48 | 0.61 | 0.57 | 0.48 | 0.59 | 0.52 | 0.42 | 0.57 |
|  | Pure PET | 0.11 | 0.15 | 0.52 | 0.11 | 0.22 | 0.50 | 0.12 | 0.23 | 0.51 |
|  | Pure AET | 0.26 | 0.06 | 0.08 | 0.23 | 0.02 | 0.07 | 0.18 | 0.01 | 0.04 |
|  | Overlap | 0.22 | 0.27 | 0.00 | 0.23 | 0.24 | 0.03 | 0.23 | 0.18 | 0.03 |
|  | Residuals | 0.41 | 0.52 | 0.39 | 0.43 | 0.52 | 0.41 | 0.48 | 0.58 | 0.43 |
| Low energy region | Full model | 0.60 | 0.28 | 0.78 | 0.55 | 0.29 | 0.71 | 0.57 | 0.26 | 0.63 |
|  | Pure PET | 0.13 | 0.14 | 0.34 | 0.13 | 0.22 | 0.35 | 0.12 | 0.22 | 0.39 |
|  | Pure AET | 0.24 | 0.04 | 0.18 | 0.16 | 0.00 | 0.08 | 0.17 | -0.01 | 0.07 |
|  | Overlap | 0.24 | 0.10 | 0.27 | 0.25 | 0.07 | 0.29 | 0.28 | 0.06 | 0.17 |
|  | Residuals | 0.40 | 0.72 | 0.22 | 0.45 | 0.71 | 0.29 | 0.43 | 0.74 | 0.37 |
| High energy region | Full model | 0.62 | 0.55 | 0.32 | 0.60 | 0.56 | 0.29 | 0.48 | 0.48 | 0.24 |
|  | Pure PET | 0.04 | 0.16 | 0.11 | 0.03 | 0.19 | 0.09 | 0.04 | 0.18 | 0.07 |
|  | Pure AET | 0.20 | 0.05 | 0.02 | 0.20 | 0.03 | 0.02 | 0.13 | 0.01 | 0.01 |
|  | Overlap | 0.38 | 0.34 | 0.19 | 0.37 | 0.34 | 0.18 | 0.31 | 0.28 | 0.17 |
|  | Residuals | 0.38 | 0.45 | 0.69 | 0.40 | 0.44 | 0.71 | 0.52 | 0.52 | 0.76 |

**Table S6** The effects of plant species richness and climate (PET and AET) on vertebrate species richness in Xinjiang, China by partial regressions. The grain sizes are 0.1° × 0.1°, 0.5° × 0.5° and 1.0° × 1.0°, respectively. PET: potential evapotranspiration and AET: actual evapotranspiration.

|  |  | 0.1° × 0.1° |  | 0.5° × 0.5° |  | 1.0° × 1.0° |  |
| --- | --- | --- | --- | --- | --- | --- | --- |
|  |  | Mammal richness | Bird richness | Mammal richness | Bird richness | Mammal richness | Bird richness |
| Whole region | Full model | 0.66 | 0.70 | 0.68 | 0.68 | 0.62 | 0.67 |
|  | Pure plant | 0.19 | 0.09 | 0.20 | 0.08 | 0.20 | 0.10 |
|  | Pure climate | 0.12 | 0.31 | 0.13 | 0.29 | 0.11 | 0.30 |
|  | Overlap | 0.35 | 0.30 | 0.35 | 0.30 | 0.31 | 0.28 |
|  | Residuals | 0.34 | 0.30 | 0.33 | 0.33 | 0.38 | 0.33 |
| Low energy region | Full model | 0.48 | 0.85 | 0.50 | 0.82 | 0.59 | 0.76 |
|  | Pure plant | 0.20 | 0.07 | 0.21 | 0.10 | 0.32 | 0.13 |
|  | Pure climate | 0.03 | 0.14 | 0.07 | 0.20 | 0.11 | 0.19 |
|  | Overlap | 0.25 | 0.64 | 0.22 | 0.52 | 0.15 | 0.44 |
|  | Residuals | 0.52 | 0.16 | 0.50 | 0.18 | 0.41 | 0.24 |
| High energy region | Full model | 0.71 | 0.45 | 0.74 | 0.42 | 0.63 | 0.36 |
|  | Pure plant | 0.16 | 0.15 | 0.17 | 0.13 | 0.16 | 0.12 |
|  | Pure climate | 0.07 | 0.03 | 0.10 | 0.03 | 0.09 | 0.01 |
|  | Overlap | 0.48 | 0.27 | 0.47 | 0.26 | 0.39 | 0.24 |
|  | Residuals | 0.30 | 0.55 | 0.26 | 0.58 | 0.37 | 0.64 |

**Figure S7** Spatial correlograms (estimated by Moran’s I coefficients) for the patterns in species richness and the model residuals. (a), plants; (b), mammals; (c), birds. In each figure, ‘Climate’ indicates residuals of climate model with PET and AET; ‘Water’ indicates residuals of water model with AET; ‘Energy’ indicates residuals of energy model with PET. In b & c, the series C & P indicates the residuals of two climatic variables and plant species richness.
